# Supplementary material for: A hierarchical transcription factor cascade regulates enteroendocrine cell diversity and plasticity in Drosophila
Source: Nat Commun. 2022 Oct 31;13:6525. doi: 10.1038/s41467-022-34270-0 (PMC9622890; doi:10.1038/s41467-022-34270-0)
Supplement: Supplementary file 1 — Supplementary Information [file 41467_2022_34270_MOESM1_ESM.pdf]

Supplemental Information

A hierarchical transcription factor cascade regulates enteroendocrine cell diversity and plasticity in *Drosophila*

Xingting Guo[1,2], Yongchao Zhang[1,2], Huanwei Huang[1,2], Rongwen Xi[1,2*]

[1] National Institute of Biological Sciences, No. 7 Science Park Road, Zhongguancun Life Science Park, Beijing 102206, China.

[2] Tsinghua Institute of Multidisciplinary Biomedical Research, Tsinghua University, Beijing, 102206, China.

* Correspondence: xirongwen@nibs.ac.cn

Supplemental figures

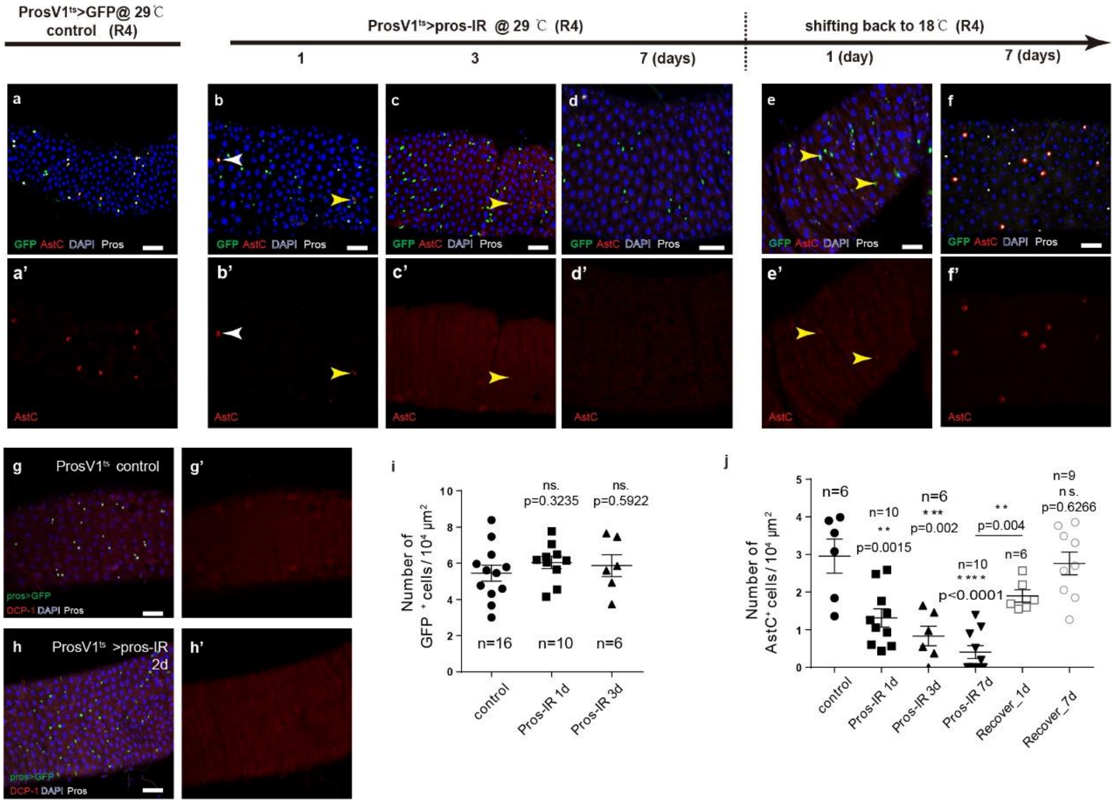

Supplemental Figure 1. A gradual loss of AstC expression following the conditional *pros* depletion in EEs. Related to Figure 1.

(a-d) Compared to normal EEs (a), knocking down *pros* for 1d (b), 3d (c) and 7d (d) lead to gradual loss of both Pros and AstC expression. During this process, a higher level of Pros expression in an EE is frequently accompanied by a higher level of AstC expression (white arrow in b vs. yellow arrows in b and c);

(e-f) The restoration of Pros expression in Pros-depleted EEs by shifting flies back to permissive temperature allows gradual re-appearance of AstC expression;

(g-h) The apoptosis marker DCP-1 staining in normal (g) and pros-depleted guts (h). No significant DCP-1 signal could be detected upon pros-depletion.

(i) Quantification of GFP^+ cells at early time (1d and 3d) of pros-depletion in EE cells showed no significant change compared with normal guts. Error bars represent Mean ± SEM; ns, not

significant (Two tailed Student’s t test); "n” indicate the number of guts used for quantification; Source data are provided as a Source Data file.

(j) Quantification of $AstC^+$ cells in the intestinal epithelium in normal, *pros*-depleted (1d, 3d, 7d) and pros-restored intestines, Mean ± SEM, ns, not significant; **p < 0. 01; ***p < 0. 001; ****p < 0. 0001 (Two tailed one way anova test); "n” indicate the number of guts used for quantification. Source data are provided as a Source Data file.

Scale bars, 50 μm

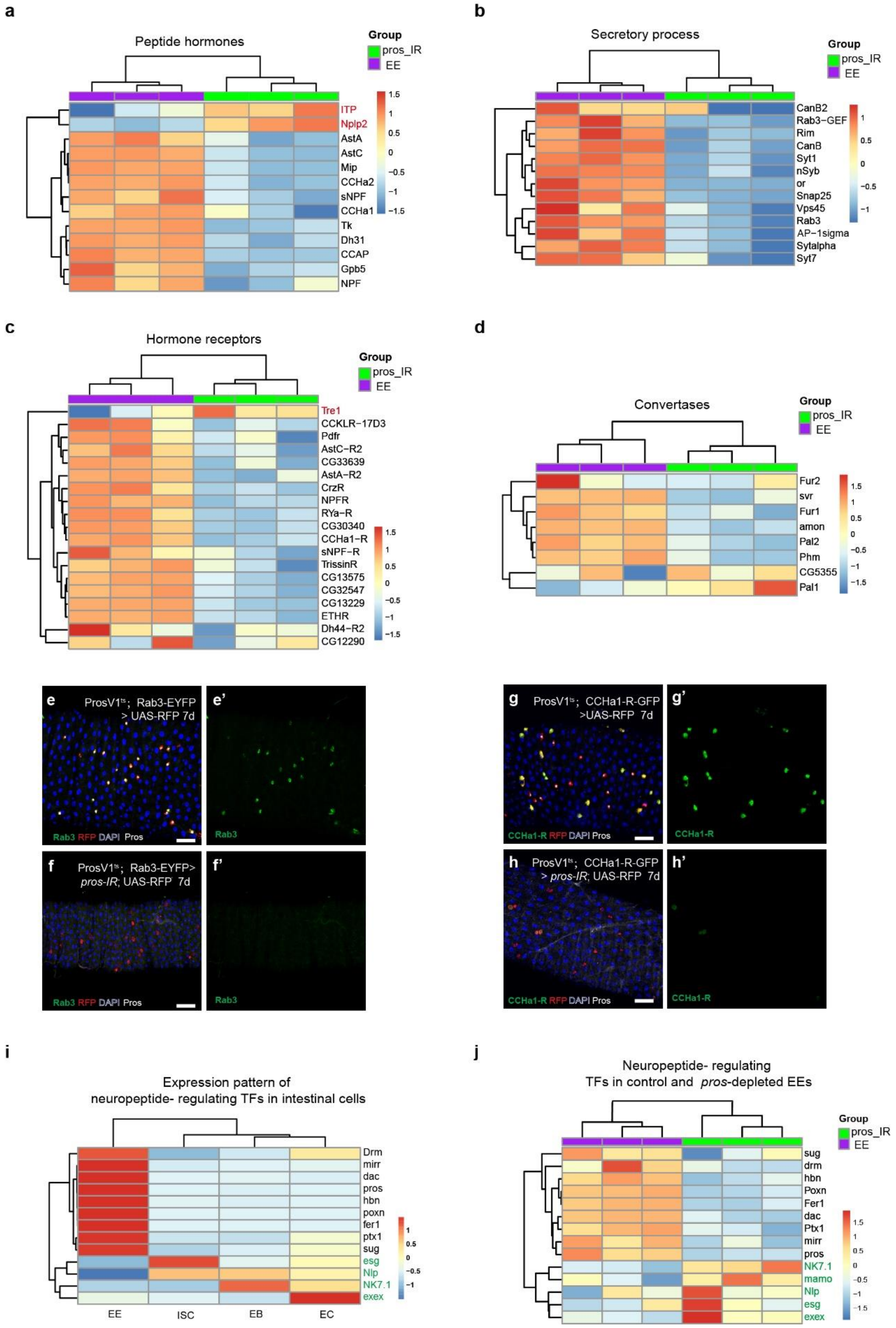
a
Peptide hormones
Group
pros_IR
EE
ITP
Nplp2
AstA
AstC
Mip
CCHa2
sNPF
CCHa1
Tk
Dh31
CCAP
Gpb5
NPF
b
Secretory process
CanB2
Rab3-GEF
Rim
CanB
Syt1
nSyb
or
Snap25
Vps45
Rab3
AP-1sigma
Sytalpha
Syt7
c
Hormone receptors
Tre1
CCKLR-17D3
Pdfr
AstC-R2
CG33639
AstA-R2
CrzR
NPFR
RYa-R
CG30340
CCHa1-R
sNPF-R
TrissinR
CG13575
CG32547
CG13229
ETHR
Dh44-R2
CG12290
d
Convertases
Fur2
svr
Fur1
amon
Pal2
Phm
CG5355
Pal1
e
ProsV1ts; Rab3-EYFP > UAS-RFP 7d
Rab3 RFP DAPI Pros
e'
Rab3
f
ProsV1ts; Rab3-EYFP> pros-IR; UAS-RFP 7d
f'
g
ProsV1ts; CCHa1-R-GFP >UAS-RFP 7d
CCHa1-R RFP DAPI Pros
g'
CCHa1-R
h
ProsV1ts; CCHa1-R-GFP > pros-IR; UAS-RFP 7d
h'
i
Expression pattern of neuropeptide- regulating TFs in intestinal cells
Drm
mirr
dac
pros
hbn
poxn
fer1
ptx1
sug
esg
Nlp
NK7.1
exex
EE
ISC
EB
EC
j
Neuropeptide- regulating TFs in control and pros-depleted EEs
sug
drm
hbn
Poxn
Fer1
dac
Ptx1
mirr
pros
NK7.1
mamo
Nlp
esg
exex

Supplemental Figure 2. Depletion of *pros* in the differentiated EEs abolishes EE identity gene expression in all EE subtypes. Related to Figure 1.

(a) A heatmap showing the transcriptional changes of peptide hormones expressed in distinct EE subtypes following *pros* depletion. The majority of peptide hormones are significantly downregulated, e.g., DH31, AstA, and sNPF. Two hormones ITP, Nplp2, which are not EE-specific, are upregulated after *pros-IR* (highlighted in red);

(b) A heatmap showing the transcriptional changes of genes involved in the secretory process in EEs following *pros* depletion;

(c) A heatmap showing the transcriptional changes of peptide hormone receptors in EEs following *pros* depletion. The majority of the receptors are significantly downregulated except Tre1 (highlighted in red), whose expression in the intestinal epithelium is not restricted to EEs;

(d) A heatmap showing the transcriptional changes of convertases in EEs following *pros* depletion.

(e-f) Rab3 is an EE-specific marker (e). Knocking down *pros* using ProsV1^{ts} > pros-RNAi causes the loss of Rab3 expression (f);

(g-h) *CCHa1-R* is normally expressed in subsets of EEs in the anterior and middle midguts (g), and knocking down *pros* using ProsV1^{ts} > pros-RNAi abolishes its expression (h);

(i-j) A heatmap showing the expression of EE-subtype-regulating TFs in different epithelial types in the intestinal epithelium (i), and in control and *pros*-depleted EEs (j). EE-specific TFs are generally downregulated upon *pros* depletion (black), but TFs that are not EE-specific shows upregulation *pros* depletion (highlighted in green).

Scale bars, 50 μm

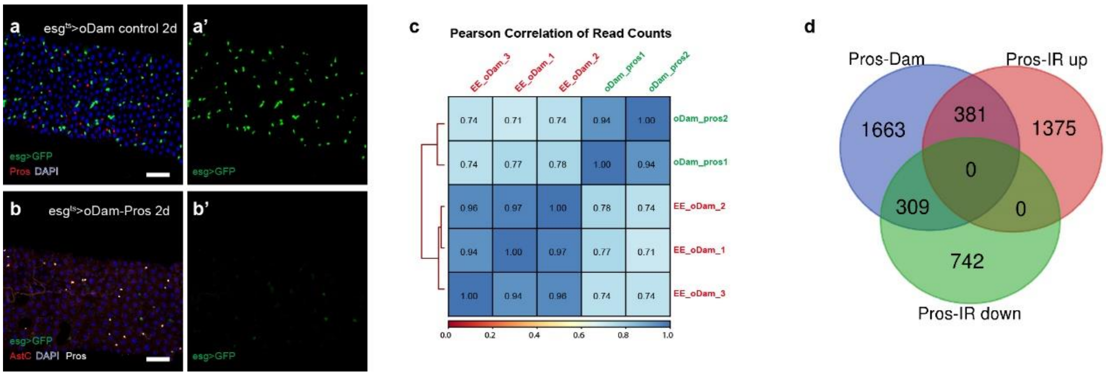

Supplemental Figure 3. Gene enrichment analysis of Pros-Dam target genes and Pros-regulating genes. Related to Figure 2.

(a-b) Test the ability of oDam-Pros transgene in inducing intestinal stem cell differentiation. Compared to normal guts (a), expressing oDam-Pros in progenitor cells using esg-Gal4 leads to rapid loss of GFP^+ cells (b);

(c) Pearson co-relation coefficient analysis of control Dam and oDam-Pros replicates;

(d) A venn diagram showing numbers of Pros-Dam target genes that are significantly up or down regulated in *pros*-depleted EEs.

Scale bars, 50 μm

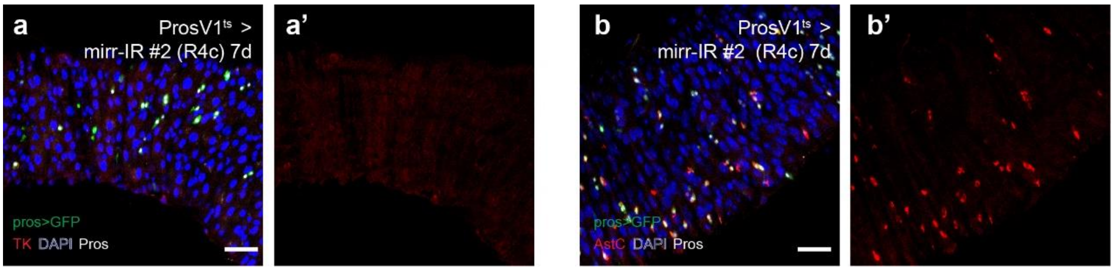

Supplemental Figure 4. Mirr oppositely regulates Tk and AstC expression. Related to Figure 3.

Knocking down mirr using another mirr-RNAi line (SH05171.N) also leads to significant down regulation of Tk expression (a), and upregulation of $AstC^+$ EEs (b)

Scale bars, 50 μm

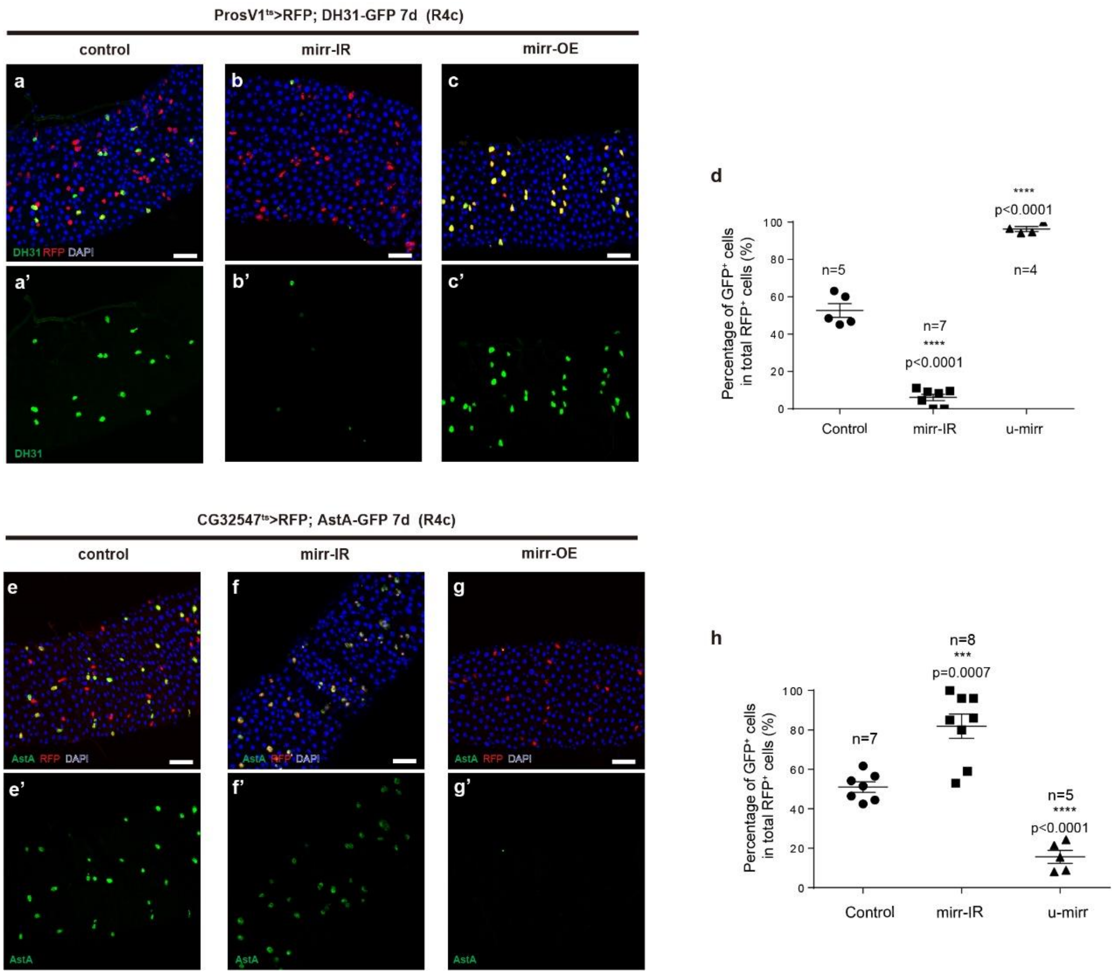

Supplemental Figure 5. Role of the 2^0 TF mirr on class level EE identity maintenance. Related to Figure 3.

(a-c) Compared to control guts (a), knocking down *mirr* in EEs leads to loss of DH31 expression in all EEs (b), whereas overexpressing *mirr* leads to gain of DH31 expression in all EEs (c);

(d) Quantification of the percentages of DH31$^+$ cells among all GFP$^+$ cells in control, *mirr-IR*, and *u-mirr* guts. Error bars indicate Mean ± SEM, ****p < 0. 0001 (Two tailed Student's t test); "n" indicate the number of guts used for quantification; Source data are provided as a Source Data file;

(e-g) Compared to control guts (e), knocking down *mirr* leads to AstA expression in all EEs (R5 region) (f), whereas overexpressing *mirr* causes the loss of AstA expression in all EE cells(g).

(h) Quantification of the percentages of $AstA^+$ cells among all GFP^+ cells in *control*, *mirr-IR*, and *u-mirr* guts. Error bars indicate Mean ± SEM, ***p < 0. 001, ****p < 0. 0001 (Two tailed Student’s t test); "n” indicate the number of guts used for quantification; Source data are provided as a Source Data file;

Scale bars, 50 μm

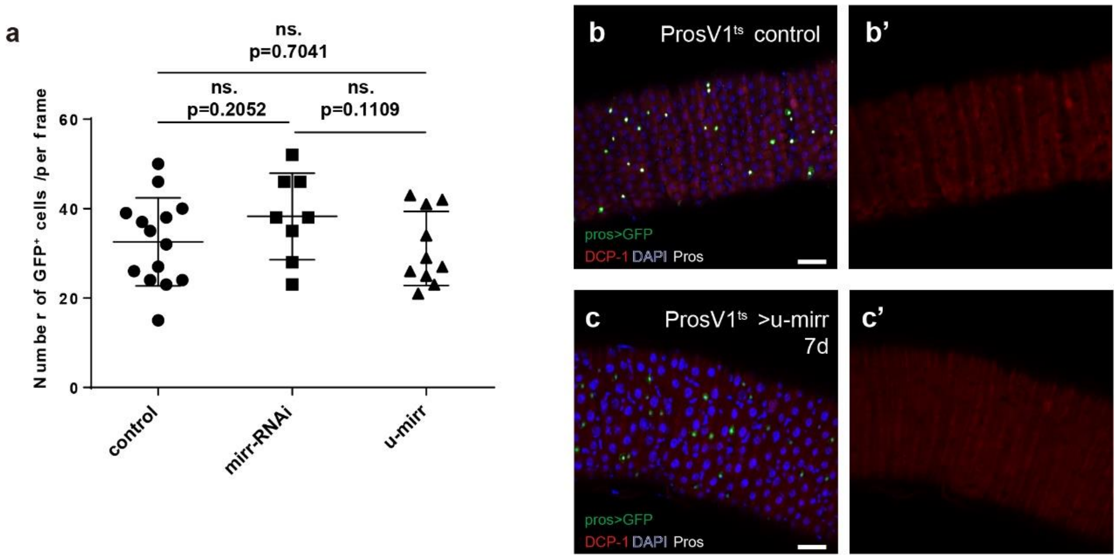

Supplemental Figure 6. Mirr activation in EEs does not cause cell apoptosis. Related to Figure 3.

(a) Quantification of GFP^+ EEs in control, mirr-RNAi and u-mirr guts. No significant changes of GFP^+ cell number is observed; Error bars indicate Mean ± SEM, ns, not significant (Two tailed Student's t test); Source data are provided as a Source Data file;

(b-c) Staining of the apoptotic marker Dcp-1 in normal (b) and mirr overexpressed EEs (c). There is no significant increase of Dcp-1^+ EEs in mirr overexpressed guts compared with control.

Scale bars, 50 μm

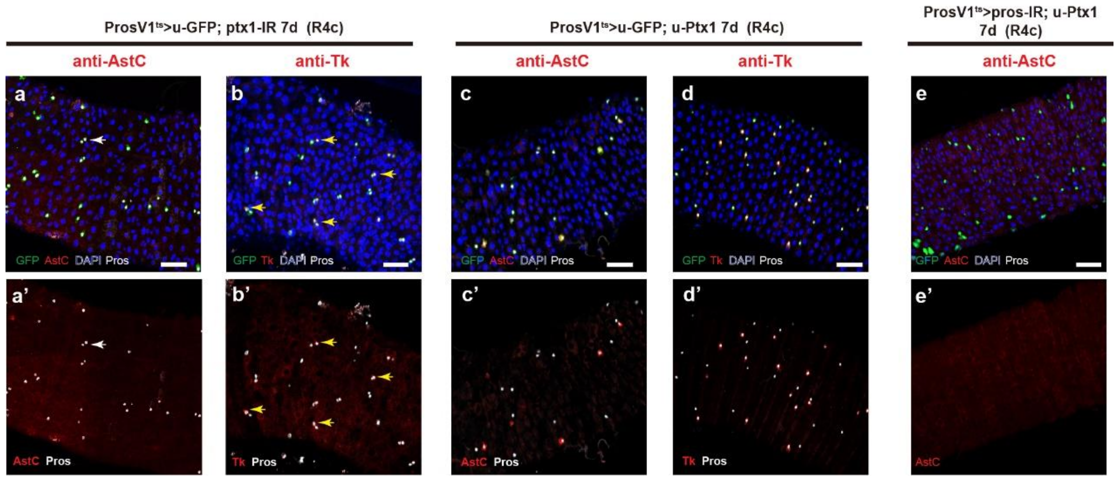

Supplemental Figure 7. Role of the 2^0 TF Ptx1 on class level EE identity maintenance. Related to Figure 3.

(a-b) Knocking down *Ptx1* in EEs leads to loss of AstC expression (R4C region) (a), without obvious effect on Tk expression (b);

(c-d) *Ptx1* overexpression in EEs has no obvious effect on either AstC (c), or Tk expression in R4C region (d);

(e) AstC expression is lost in *pros*-depleted EEs despite *Ptx1* is overexpressed;

Scale bars, 50 μm

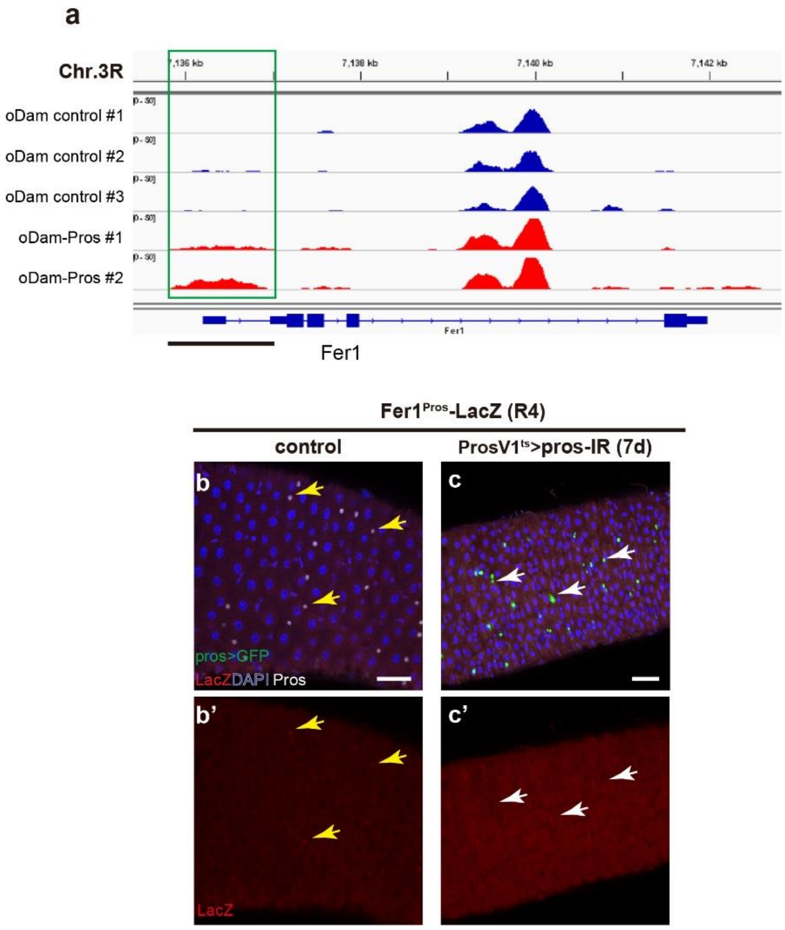

Supplemental Figure 8. The regulatory role of Pros on the 3^0 TF Fer1. Related to Figure 5.

(a) A Pros-DamID binding region (frame outlined) is found at the Fer1 locus;

(b-c) A LacZ reporter driven by the putative Pros binding sequence on Fer1 locus (denoted by the black line) marks a subset of EEs located at R4 region (b, yellow arrow heads). Knocking down of *pros* abolishes this reporter expression (c, white arrow heads),

Scale bars, 50 μm

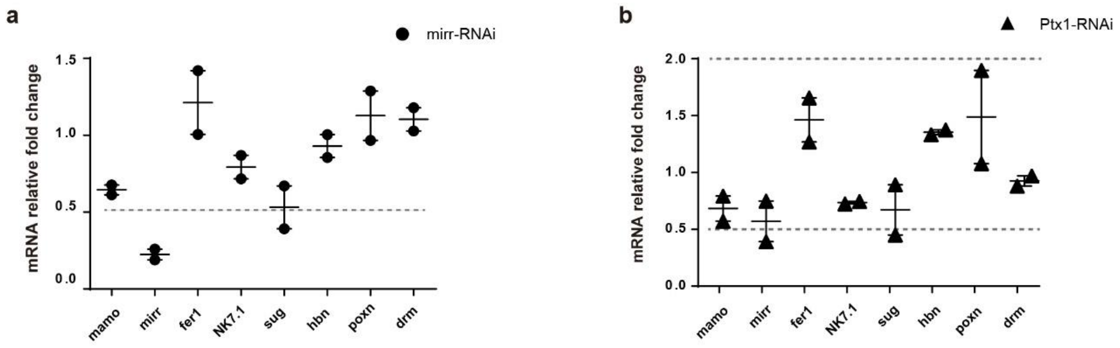

Supplemental Figure 9. The 2^0 TFs (mirr and Ptx1) does not appear to regulate the expression of the 3^0 TFs. Related to Figure 5.

(a-b) qPCR analysis of the transcriptional changes of 3^0 TFs following the depletion of the 2^0 TF *mirr* (a) or *ptx1*(b) in EEs driven by ProsV1-Gal4^{ts}; data are plotted as mean relative fold-changes ± SEM, reactions were performed in triplicate on two independent biological replicates (n=2).

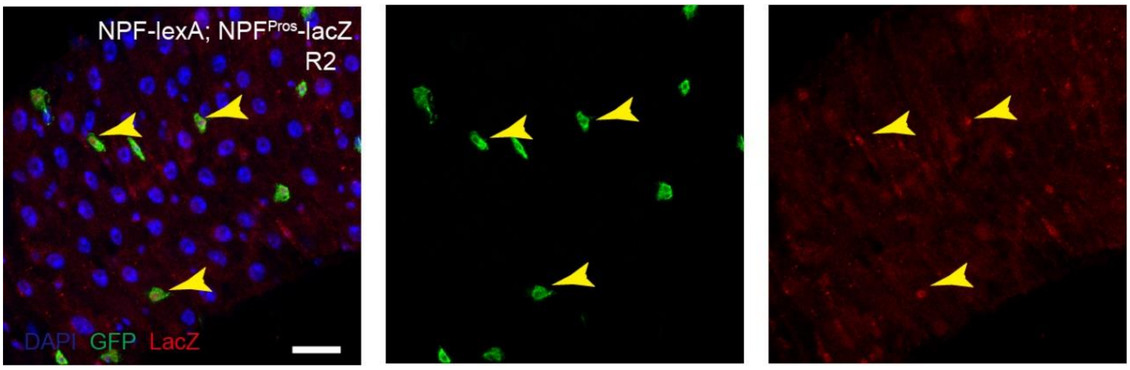

Supplemental Figure 10. Characterization of CCHa1^{Pros}-lacZ expression. Related to Figure 5.

The expression of NPF^{Pros}-lacZ is largely overlapped with the expression of NPF-lexA> lexAop-GFP (yellow arrow heads).

Scale bar, 50 μm

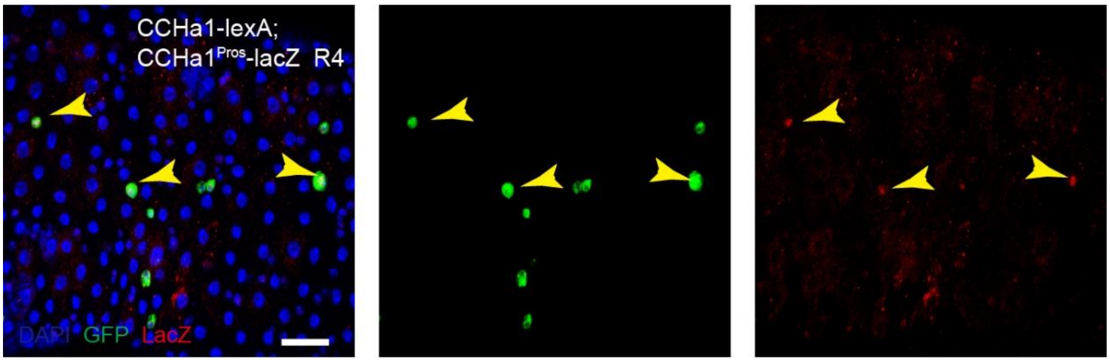

Supplemental Figure 11. Characterization of CCHa1^{Pros}-lacZ expression. Related to Figure 5 and 6.

The expression of CCHa1^{Pros}-lacZ is found in a subset of CCHa1-lexA>lexAop-GFP cells (yellow arrow heads).

Scale bar, 50 µm

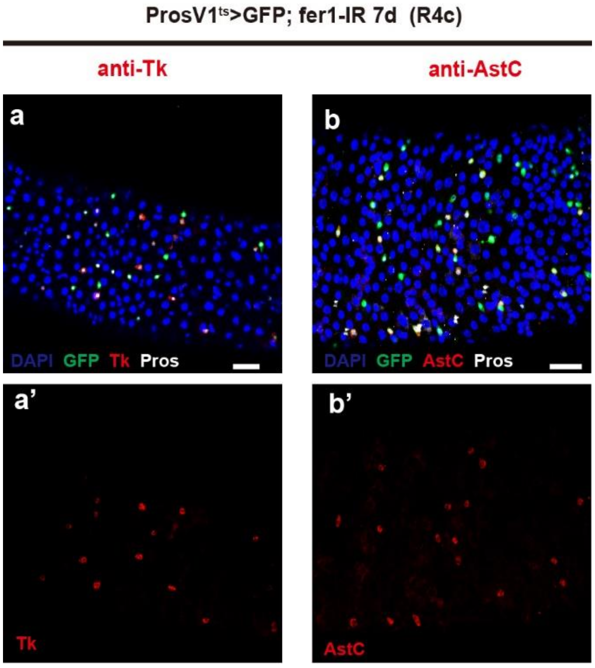

Supplemental Figure 12. The requirement of Fer1 in Tk or AstC expression. Related to Figure 6.

Knocking down the 3^0 TF *fer1* does not have any obvious effect on the expression of Tk (a) or AstC (b);

Scale bars, 50 μm

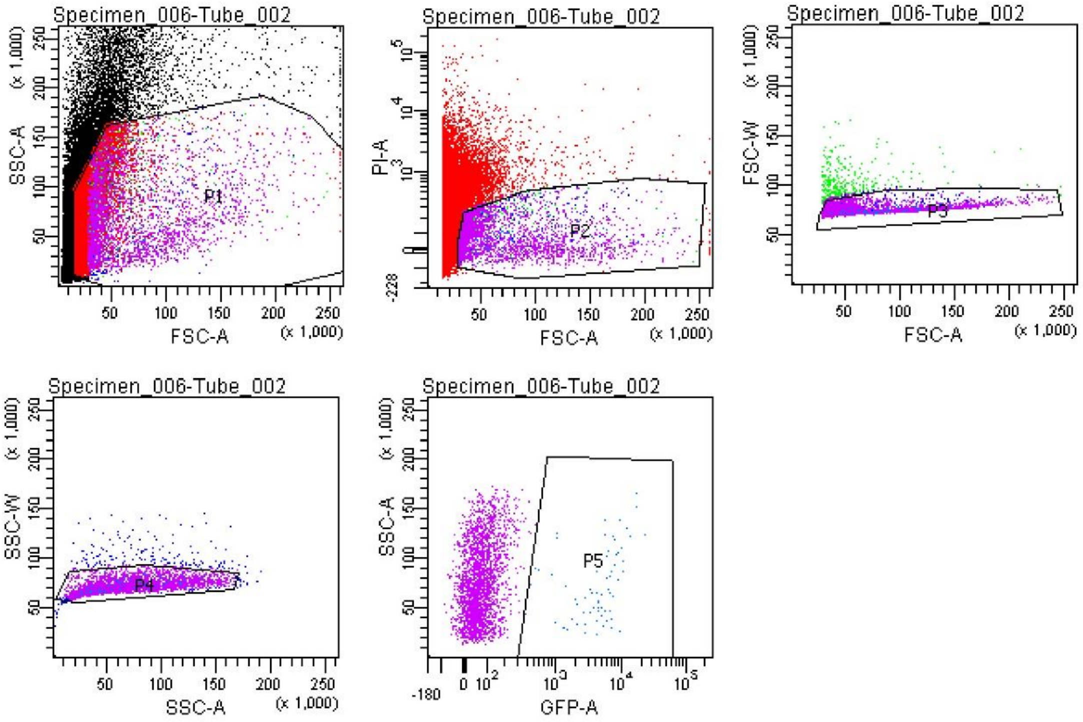

Population	#Events	%Parent	%Total
All Events	100,000	####	100.0
P1	25,328	25.3	25.3
P2	3,051	12.0	3.1
P3	2,765	90.6	2.8
P4	2,559	92.5	2.6
P5	70	2.7	0.1

Supplemental Figure 13. FACS sequential gating/sorting strategies to sort ProsV1-Gal4^{ts} > GFP^{+} cells in normal and *pros*-depleted guts. GFP^{+} cells in P5 were sorted and used for RNA-sequencing. Related to Figure 1 j-l.
