# Supplementary material for: A hierarchical transcription factor cascade regulates enteroendocrine cell diversity and plasticity in Drosophila
Source: Nat Commun. 2022 Oct 31;13:6525. doi: 10.1038/s41467-022-34270-0 (PMC9622890; doi:10.1038/s41467-022-34270-0)
Supplement: Supplementary file 3 — Description of Additional Supplementary Files [file 41467_2022_34270_MOESM3_ESM.pdf]

Description of Additional Supplementary Information Files Document

Supplemental data1. Significantly up and down regulated genes (sheet1) and gene hits for peptides, receptors, secretory process and TFs (sheet2) in pros-depleted EE cells. Related to figure 1j.

Supplemental data 2. Gene symbols of top 250 EE identity genes. Related to Figure 1k

Supplemental data 3. GO analysis of significantly down-regulated genes in pros-IR cells. Related to Figure 1l.

Supplemental data 4. List of oDamPros targeted genes among the top 250 EE identity genes. Related to figure 2b.

Supplemental data 5. List of genes that are both oDam-Pros targets and significantly down regulated in pros-IR EE cells (sheet 1), and GO analysis of these genes (sheet 2). Related to Figure 2c.

Supplemental data 6. Gene hits for peptides, receptors, secretory process and TFs both in Dam-ID and RNA-seq. Related to figure 2d.

Supplemental data 7. qPCR primers for EE subtype regulating TF. Related to Figure S9.
